# Nanosurgical and Bioengineering Treatment of Human Anterior Cruciate Ligament Tears with Ultrasound-Guided Injection of Modified Platelet-Rich Plasma Using Human Cell Memory Based on Clinical, Ultrasound, MRI, and Nanoscope Analyses: A Double-Blind Randomized Trial

**DOI:** 10.3390/jcm13092475

**Published:** 2024-04-24

**Authors:** Cezary Wasilczyk

**Affiliations:** Medical Department, Wasilczyk Medical Clinic, ul. Kosiarzy 37/80, 02-953 Warszawa, Poland; wasilczyk.chirurg@gmail.com

**Keywords:** anterior cruciate ligament tears treatment, ACL, nanosurgery and bioengineering treatment

## Abstract

**Background**: Anterior cruciate ligament (ACL) tears account for 40% to 50% of all ligamentous knee injuries. Most patients with ACL ruptures undergo surgical treatment. There is currently no objective, well-documented, repeatable, and standardized nonsurgical method for ACL tear treatment. This study aimed to investigate ACL outcomes in patients who underwent a novel nanosurgery and bioengineering treatment (NSBT) for an ACL tear. **Methods**: This was a double-blind randomized trial including 44 patients with a history of traumatic knee injury and a confirmed ACL tear. The final sample comprised 40 patients who met all the eligibility criteria. The patients were divided into two groups: the treatment group (n = 30) and the control group (n = 10). The treatment group underwent nanosurgery with an ultrasound-guided injection of modified platelet-rich plasma (PRP) using human cell memory (RP-hCM). The control group was treated with an ultrasound-guided PRP injection into the joint capsule. At baseline and post-treatment, all patients underwent both ultrasonography and magnetic resonance imaging (MRI), and the following clinical variables were assessed: the WOMAC score, the Lysholm knee score, the visual analog scale score, and knee instability. In most patients, the clinical outcome was verified using nanoscopy. **Results**: The median WOMAC, VAS, and LKS scores, as well as knee instability, improved significantly 12 weeks after the procedure in the treatment group (*p* < 0.001). We found a significantly larger improvement in the assessed parameters in the treatment group compared to the control group (*p* < 0.001). In the treatment group, all the patients had good and very good clinical outcomes, while 90% of the patients had a normal ACL signal in a follow-up MRI scan. In the control group, a physical examination revealed no changes in knee stability after treatment. **Conclusions**: This study showed that there is a significant difference in patient experience and the duration of recovery for patients with ACL tears treated with NSBT. The novel nonsurgical method was shown to be repeatable, objective, well documented, standardized, and highly effective.

## 1. Introduction

Anterior cruciate ligament (ACL) tears account for 40% to 50% of all ligamentous knee injuries, and because of the worst healing percentage of all ligaments in the human knee, they are also the most challenging to treat. The anterior cruciate ligament is responsible for the stabilization of the human knee in rotational and anterior movements, along with its derivatives. Instability of the knee due to an ACL tear leads to rotational and anterolateral instability of the knee, meniscal and chondral lesions, and, finally, osteoarthritis (OA) [1,2]. In recent years, scientific research has noted a gradual increase in ligamentous injuries, including the ACL, due to increasing interest in sports activities.

In most cases of total or partial ACL tears, classical reconstructive surgery is recommended [3,4]. In conservative, non-operative methods, some authors propose immobilization, rehabilitation, including strengthening and stretching the muscles, proprioception training, and bio-orthopedic treatment. As mentioned above, reconstructive knee procedures for an ACL tear are currently the most effective and highly recommended treatment [2,5,6]. Recent trials have revealed many new intraoperative techniques for improving treatment outcomes [7].

ACL injury division may depend on clinical and imaging findings or on arthroscopic assessment. The division of structural ACL injuries may depend on the following: (1) being a total or partial tear (with a subdivision of tears that are anteromedial, posterolateral, or both); (2) a proximal, distal, or intra-fiber rapture; and (3) an isolated or concomitant ACL injury—multi-ligamentous, meniscal, and osteochondral injuries [3]. Noyes defined total ACL tears as those in which over 75% of ligament fibers are torn [8]. Also, Hong et al. defined partial ACL tears depending on the percentage of ACL fiber torn. According to the author, a partial ACL injury is defined as less than 50% being torn [9]. The diagnosis of partial tears of the ACL is still controversial and challenging for orthopedic surgeons. The incidence of partial tears ranges from 9% to over 27% [3]. So far, the difference in definition between a partial or total ACL tear is unclear. Some define ACL tears based on operational findings, while others define them based on clinical or imaging evaluations. The American Medical Association divides ACL injuries into three degrees: the first and second degrees are defined as a partial tear, and the third degree is defined as a complete tear of the ACL [3].

For non-operative treatment methods, some authors propose immobilization, rehabilitation, including strengthening and stretching the muscles, and proprioception training. Although there are attempts in the literature to biologically treat ACL tears, there is no proven, repetitive, standardized method of bioengineering. In the case of non-operative treatment failure, the surgeon may change the qualification to operative techniques [3]. In arthroscopic–reconstructive methods, the surgeon makes a decision on the treatment options in a selective way. Postoperative treatment and rehabilitation take from 6 to 9 months [10].

A patient qualifying for ACL reconstructive surgery must be admitted to a hospital, and the operative procedure must be performed in an operating room. Postoperative recovery takes weeks to months, and surgery does not guarantee excellent clinical outcomes. Operative techniques may entail local or general short- or long-term complications. Although there are cases of spontaneous ACL healing noticed in the literature review [11,12,13], there is no well-documented, repeatable, and highly effective, standardized, non-operative, bioengineering technique for ACL tear treatment.

In the presented study, we evaluated the effectiveness of a modified PRP treatment that includes growth hormones—somatotropin and strophanthus combe—in appropriate doses. Somatotropin enhances the anabolic effect on tissues, while strophanthus combe, a drug from the group of glycosides, inhibits the action of the sodium–potassium pump. This inhibition increases the concentration of sodium and calcium within the cell, thereby enhancing cellular turgor. The action of glycosides on the sympathetic–parasympathetic nervous system has also been proven to increase the sensitivity of cellular receptors, including baroreceptors, in response to injury [14,15]. Increasing cellular turgor and anabolic processes directly activate cellular memory. Together with precisely selected injection sites, this combination begins to regenerate the injured ACL. The primary objective of this study was to assess ACL outcomes in patients with a history of traumatic knee injury and an ACL tear confirmed by clinical evaluation and imaging studies, including ultrasonography, magnetic resonance imaging (MRI), and nanoscopy, who underwent nanosurgery with an ultrasound-guided injection of modified platelet-rich plasma (PRP) using human cell memory (RP-hCM). In addition, the aim of the study was to objectify and standardize this novel method of ACL tear treatment.

## 2. Materials and Methods

### 2.1. Study Design

This double-blind randomized study (ISRCTN15642019) was conducted between March 2015 and February 2024 and included consecutive patients with ACL tears confirmed by MRI and ultrasonography. The inclusion criteria for the study were as follows: a history of traumatic knee injury, positive Lachman and pivot shift tests upon physical examination, an ACL tear confirmed by ultrasonography and MRI, no coexisting knee injuries requiring surgery, and written informed consent to participate in the trial. The exclusion criteria for the trial were pregnancy and the absence of written informed consent.

Due to ethical considerations, the study was designed with unequal group sizes—anticipating an expected improvement of 80% in the experimental group based on our experience and 10% in the control group, which aligns with studies that have proven that PRP does not have a regenerative effect on the ACL. There was a common standard deviation of 20% for 30 patients in the experimental group and 10 in the control group, yielding a test power greater than 0.99. Patients were assigned in a 3:1 ratio to the treatment or control group. A simple randomization model was used, with sequentially numbered, opaque, and sealed envelopes to conceal the allocation. Patients were blinded to which treatment group they were assigned and were unblinded at a 6-week follow-up visit. Data collectors and assessors were also blinded. An independent examiner was blinded to the nanosurgical and injection sides and the study group.

The study was approved by the Bioethical Committee in Warsaw, Poland (No. 52/21 from 18 November 2021). All procedures were performed in compliance with the Declaration of Helsinki.

### 2.2. Intervention

Patients in the treatment group underwent nanosurgery and bioengineering procedures with an ultrasound-guided RP-hCM (modified form of PRP) injection directly into the core points in the ACL fiber structure joint capsule. The determination of the core points is crucial in this technique and is carried out through ultrasound, MRI, and clinical evaluations. In the control group, patients received an ultrasound-guided injection of PRP into the internal space of the joint capsule without determining the core points. Based on the literature, a PRP injection inside the joint capsule has no effect on ACL healing [16,17,18].

The RP-hCM injection was prepared using PRP obtained through the centrifugation (1800 rpm/min for 8 min) of 6–10 mL of patient blood and aseptically adding growth hormones—somatotropin and strophanthus combe—in appropriate doses. The method of RP-hCM and PRP preparation was standardized. All nanosurgery procedures were also standardized and performed under local anesthesia in sterile conditions in an ambulatory setting. Patients were placed in a stomach position using posterior access. ACL fibers were defined by ultrasonography. Using a needle (0.6–0.8 mm × 70–80 mm) and ultrasound guidance, a percutaneous injection of RP-hCM was injected into the joint capsule directly into ruptured ACL fibers. All patients underwent 1 to 3 nanosurgeries with injections of RP-hCM. The number of injections was tailored to each individual patient and depended on clinical and imaging evaluations. The post-treatment protocol was standardized for all patients.

The criteria for recovery were as follows: negative clinical tests of ACL failure; normal ACL course and signal upon ultrasound and MRI examinations; and no patient complaints or knee disorders during daily and sports activities according to the same training schedule as before the ACL injury.

### 2.3. Outcomes

The study protocol for all patients was standardized and included clinical analysis at baseline and after treatment. The following clinical variables were assessed at baseline and after treatment: the WOMAC score, the visual analogue scale (VAS) score, the Lysholm knee score (LKS), and knee instability. These scales were chosen due to their proven reliability and validity [19,20]. The physical examination was based on the results of the Lachman test and pivot shift test performed, with a score of 0 to 4 points for each. The maximum score of 8 points indicated, by physical examination, firm clinical instability. Examinations were performed on the day before the procedure (baseline) and 24 h, 48 h, and 6, 10, and 12 weeks after the procedure.

### 2.4. Imaging Examination

The standardized MR imaging protocol consisted of sagittal, coronal, and axial sequences, which are viewing planes that help determine the ACL structure. There are many studies that have proven the high sensitivity and specificity of MRI based on arthroscopy findings [21,22,23,24,25,26]. In our study, MRI examination played a crucial role in determining and comparing the ACL structure before and after treatment.

### 2.5. Statistical Analysis

The aim of the statistical analysis was to assess whether there were significant differences in the outcomes between baseline and follow-up in the treatment group and whether there were significant differences in the outcomes between the treatment group and the control group. Analyses were based on data obtained at baseline and after 12 weeks of treatment for the WOMAC, the VAS, the LKS, and knee instability. As all the above-mentioned quantities are ordinal values, nonparametrical tests were used. Differences between 2 measurements of the same value were assessed using a repeated-measures Wilcoxon test. For a comparison of all the numerical variables between the study group and the control group, a Mann–Whitney U test, Student’s *t*-test, or chi-squared test was used. The threshold level for statistical significance was set at 0.05. All analyses were performed using Statistica 13.3 software (Statsoft, Tulsa, OK, USA).

## 3. Results

### 3.1. Patients

**Figure 1:**
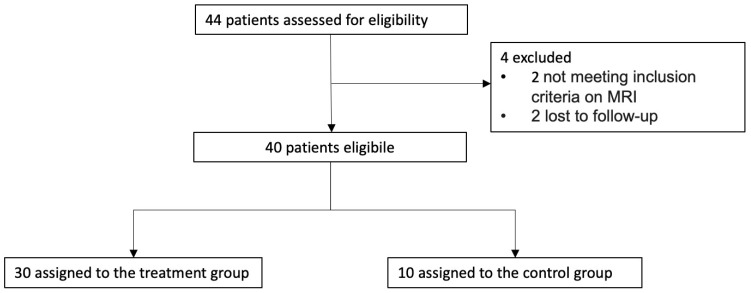
Flowchart of patient enrollment.

During the study, 44 patients were recruited and assessed for eligibility. Four patients were excluded due to not fulfilling the research criteria. Of the 44 patients, 40 were randomly assigned to the treatment group (n = 30) or the control group (n = 10) (Figure 1). All patients had ACL tears after a traumatic knee injury confirmed by clinical, ultrasound, and MRI examinations.

The patients in the treatment group were older than those in the control group (median age, 46.5 years vs. 33.0 years). The median follow-up of patients in the treatment and control groups was 54.5 months and 49.5 months, respectively. The median time from injury to the procedure was 30 days in both treatment groups. The detailed characteristics of the patients are presented in Table 1.

### 3.2. Clinical Outcomes

The patients treated with nanosurgery and RP-hCM (the treatment group) had good and very good outcomes in the clinical evaluation. The median WOMAC, VAS, and LKS scores, as well as knee instability, improved significantly 12 weeks after treatment in the treatment group (*p* < 0.001) (Table 2).

We found a significantly larger improvement in the assessed parameters in the treatment group compared to the control group (*p* < 0.001) (Table 2).

### 3.3. Imaging Analysis

In the treatment group, 27 of the 30 patients (90%) showed a normal ACL signal in the MRI scan. Nanoscopy, which was performed in 17 of the 30 patients (56.7%), revealed a healed ACL. One of the patients assessed by nanoscopy presented a proximal ACL footprint on the femoral condyle, but anteriorly to the original, according to the MRI scan posttreatment. All 30 patients after the NSBT procedure and 2 patients treated with PRP injections left the physician’s office on their own. One patient experienced temporary pain lasting 3 days, but it did not affect their regular daily activities. The remaining 29 patients did not report any complaints after treatment. At a mean follow-up of 10 weeks from baseline, 27 patients from the treatment group presented with knee stability based on their Lachman and pivot shift test results. Three patients in a post-treatment control MRI examination presented with a nonadjacent ACL, despite some improvement in the WOMAC, VAS, and LKS scales, but their knee instability in the clinical examination was not reduced. In the treatment group, follow-up MRI revealed ACL healing with normal continuous tissue, and this was confirmed by a nanoscopic view. The majority of patients in the treatment group who underwent an MRI scan within the specified time frame had a normal MRI scan at a mean time of 3.4 months (range: 1 to 12 months) from the intervention.

In the control group, the patients showed knee instability after treatment upon physical examination and a dynamic ultrasound examination. Moreover, upon follow-up MRI, the ACL presented as nonadjacent. The analysis of the control group revealed that the knee instability upon physical examination was at the same level before and after treatment; however, the analysis of the other examined variables showed some improvements. Those patients were referred for surgical reconstruction of the ACL, and a perioperative view confirmed a nonadjacent ACL.

All the MRI analyses in the treatment group, except one, revealed a healthy, intact ACL with a normal signal, as shown in Figure 2, Figure 3, Figure 4, Figure 5, Figure 6, Figure 7 and Figure 8. Figure 9 shows an example of an unregenerated ACL in a patient from the control group.

## 4. Discussion

The operative reconstruction of the ACL is the gold standard nowadays, but with this method of treatment, there are still many risks and unknown factors. In the literature, the incidence of graft rapture only ranges from 3.2 to 11.1%. Even if reconstruction surgery is successful, there are many post-surgical biomechanical alterations of the knee that may result in internal impingement that contributes to accelerating the development of OA. Also, differences in the proprioception of native and transplanted ACL in surgery procedures should be taken into consideration [27].

The study presented is the first worldwide announcement of a novel, non-operative, repeatable, objective, well-documented, standardized, highly effective, and rapid recovery method for treating human ACL tears. This method is based on the Lysholm knee scoring system and ultrasound, MRI, and clinical analyses and utilizes nanosurgery and bioengineering techniques. In this study, based on a clinical analysis including the WOMAC, Lysholm knee, and VAS scores, as well as a physical examination, the patients treated with RP-hCM showed significant differences between the baseline and follow-up outcomes. These clinical outcomes also differed significantly between the treatment and control groups. Ultrasonography, MRI, and nanoscopy confirmed good and very good outcomes in the treatment group. This study, which combined a bioengineering, anatomical, and functional approach, is the first to describe a highly effective, documented, repetitive, and standardized method of nonsurgical ACL treatment offering a rapid recovery. It is worth noting the long-term follow-up of the treatment group, with a median of 54.5 months. The procedure is performed in an ambulatory setting under local anesthesia. Hospital admission is not required, and patients can return to daily activities on the same day. The study showed that the time of recovery after bioengineering procedures depends on age, time from injury to baseline, and the patient’s involvement in rehabilitation. Importantly, one of the patients in the treatment group was an active speedway rider from the World Speedway First League. He sustained his injury in the middle of the season and returned to full performance and competition within 8 weeks, with no functional deficits. The rupture occurred in the left knee, which is the key and most heavily loaded structure in this sport. Thanks to the treatment, the patient did not miss the season and was able to continue his sports career.

In recent times, there have been many attempts to prove that bioengineering treatment is a natural method that tends to minimize surgical procedures, reduce difficulty for patients, and minimize recovery time. The primary healing potential of the ACL has been reported to be extremely poor based on clinical and experimental studies [28,29,30]. There are many studies investigating technical and biological methods of improvement to amplify ACL healing [11,12,13,28,29,30]. In the literature review, there is evidence that ACL remnants have the ability to revascularize, proliferate, and spontaneously heal.

Independent scientists, in in vitro and in vivo models of animal and human ACL remnants proved at the histological level, that PRP significantly increased cellularity, increased angiogenesis, and promoted earlier and more organized ligament filling, as well as showing its influence on the ACL [11,31,32,33].

Dhillon et al. [34] reported in their in vitro research that PRP has an enhancing effect on human ACL cell viability and promotes cell proliferation for the primary repair of ACL tears. It was a very important step forward in ACL tissue bioengineering treatment methods.

In scientifically considering ACL regeneration method research, they came to the conclusion that the fibrin clot containing the platelets may have been prematurely dissolved in the intra-articular space, leading to the failure of ACL regeneration [17,32,33].

Murray et al. reported that the treatment method of PRP intake into space between the transected ACL in a porcine model showed no evidence of ACL regeneration and healing; however, Kobayashi et al. demonstrated improving ACL fiber healing in a canine model through their increased vascularity compared with the control [17,32,35]. Kondo et al., in in vivo studies of a rabbit model, proved that the application of 4 ng of transforming growth factor beta 1 significantly enhanced regeneration in the injured anterior cruciate ligament group when compared to the controls [33].

In relation to the histological evidence of PRP’s influence on ACL remnants in in vitro studies and discrepancies in in vivo studies of animal models, some scientists proposed combining a collagen scaffold with autologous platelets containing hydrogel [36,37,38]. Following the above results, studies on animal models reported significantly improved ACL repair outcomes by combining a collagen scaffold with autologous platelets [35,36,37,38]. Simultaneously, Fleming et al. reported no significant improvement in ACL tear regeneration using a collagen scaffold alone without PRP in a porcine model [39].

In humans, there are reported and well-documented cases of the spontaneous healing of a ruptured ACL [11,12,13], but in the majority of patients treated with non-operative and biological methods, it has been reported to fail [11,12,13,31,32]. Costa-Paz et al. [40] retrospectively reviewed 14 patients with acute ACL tears. The purpose of the study was to determine if a complete ACL rupture in patients can spontaneously heal without the use of a specific rehabilitation program or bracing and if the patients are able to return to their athletic activity after spontaneous ACL healing. All patients were athletically active before their injury. Surgery was indicated in all cases but postponed. Based on clinical and MRI findings in a mean time of 30 weeks, 10 patients were totally healed, and 4 were nearly normal. All knees regained the end point in a negative pivot shift test. MRI at follow-up showed an end-to-end continuous ACL with a homogeneous signal. Roe et al. documented that 18 of 19 patients after an ACL tear were spontaneously healed in a 1-year follow-up based on MRI findings [13]. Ultrasound examination is also a highly effective tool for determining not only superficial tissues but also deep localized structures like the ACL, the PCL, and the meniscus in the knee joint. In the presented study, ultrasound examination plays the most important role in determining the core point of RP-hCM intake to the ACL [41,42].

In a systematic review of the current literature, Figueroa et al. [43] were searching for proof of whether PRP injections can be crucial in the treatment of ACL tears. They analyzed 516 patients—266 patients with ACL operative reconstruction with PRP injections and 250 without PRP injections—in 11 studies. A total of six studies showed a significant difference and a faster tendency to graft maturation; one study showed no differences in the groups of patients compared. In a tunnel healing assessment in one study, there was clinical benefit to using PRP, and in five cases, there were not. In conclusion, in analyzing ACL graft healing, there is more evidence that patient groups with the application of PRP have a tendency toward faster graft healing compared to patients without PRP injections. In their study, Dhillon et al. [34] evaluated the effect of both autologous PRP and PPP on human ACL cell growth characteristics. During operative procedures, the remnants of human ACL were taken, and then ACL cells were isolated, identified, cultured, and divided into six groups. Different concentrations of PRP (5% and 10%) and PPP were added to each group. Cell viability was assayed by MTT and Annexin V assays, and the DNA content was evaluated by propidium iodide staining and flow cytometry. The results of the analysis of the cultured cells showed that the addition of PRP (5 or 10%) increased the viability of ACL cells in 4 out of 11 donor samples and promoted cell proliferation in 8 of 11 donor samples. The difference in the effectiveness of 10% PRP was not significantly better than that of 5% PRP. In conclusion, Dhillon underlines that PRP may have an enhancing effect on ACL cell viability and the promotion of cell proliferation for the primary repair of an ACL tear. Dhillon proved that there is evidence that PRP intake is a good tool to intensify ACL graft maturation; on the other hand, there is no evidence of the benefits of using PRP in tunnel graft healing.

Cook et al. [44] used a canine model to determine the effects of multiple intra-articular injections of leuko-reduced PRP autologous conditioned plasma (ACP) compared to saline injections on anterior cruciate ligament healing, meniscal healing, and the progression of OA. Twelve dogs underwent partial ACL transection and meniscal release in one knee. After 6 months, the dogs were assessed for ACL material properties and histopathology. In the PRP-ACP-treated group, ACL histopathology was significantly (*p* < 0.05) less severe compared to saline-treated knees. The ACP-treated knees showed evidence of ACL repair and less severe synovitis. Cook et al. [44] proved that multiple leuko-reduced PRP injections used in a canine knee model significantly improved ACL histopathology healing. The control group were dogs with saline-treated knees. Also, in an arthroscopic assessment, the knees after multiple PRP injections compared to the normal saline-treated group were closer to normal. In clinical evaluations, multiple PRP injections (an average of five) intensify healing, improve the range of motion, and decrease effusion, pain, and limb dysfunction [44].

Bozynski et al. [45], in their study, investigated whether a single PRP injection to the knee joint of purpose-bred hounds for an acute ACL injury gave rise to better clinical effects compared to the standard of care and washout groups. Twenty-seven purpose-bred research hounds underwent knee surgery. Dogs were randomized into three treatment groups: standard of care (i.e., rest and nonsteroidal anti-inflammatory drugs [NSAIDs]), washout, or leuko-reduced PRP treatment. All groups were assessed over the following 8 weeks. Based on clinical and arthroscopic evaluations, in the PRP group, the most benefits in ACL healing were found.

Magarian et al. [46], in their study, looked at whether there was an age dependence of the response of human ACL fibroblasts to stimulation by PRP. Three basic parameters for wound healing were determined: cell migration, cell proliferation, and scaffold contraction. Migration and proliferation were significantly higher in immature cells, but no differences were seen in wound contraction. The search results showed that immature PRP patients had a better response than adolescents in terms of ACL regeneration, and this was an important step forward in ACL tissue bioengineering.

Koch et al. [47] showed that intraoperative intraligament ACP intake gave rise to good-to-excellent results based on clinical and functional evaluations in a mean 33-month follow-up.

Recently, Seijas et al. [48] revealed very good clinical outcomes in partial ACL tears treated with PRP intake during an intraoperative procedure. In a group of 19 football players, only 1 was not able to return to the sport. In the recovery group, 3 patients got back to sport in an average of 12 weeks, and the other 10 patients did so in an average of 16 weeks.

Centeno et al. [49] proposed a percutaneous injection of autologous bone marrow concentrate and platelet products in patients with symptomatic ACL tears. The injections were performed using fluoroscopy to guide the needle placement. Based on pre- and post-treatment assessments using the Numerical Pain Scale, the Lower Extremity Functional Scale, the International Knee Documentation Committee form, and MRI examination analysis, there were significantly different treatment outcomes in the patient groups pre- and post-treatment. Lee-Barthel et al. [50], in their experimental methodology study, presented an approach in which human cells isolated from ACL tissue remnants, combined with exercise-conditioned serum expanded in a culture, may be used to form a platform of engineered human ligaments. The tissue engineering model presented by the authors was used to investigate the anatomical and clinical research questions in this study.

Although Koch et al. [47] presented good and excellent treatment results for ACP injections to the ACL, the study outcomes were defined mainly through clinical assessment without an MRI follow-up examination, and all admissions were performed during intraoperative procedures. Centeno et al. [49] first proposed the percutaneous procedure of injecting biological agents into ACL remnants; however, this method was not standardized, and the author of the research in conclusion stated that all MRI examination changes could be the result of scar tissue formation rather than healing in the ACL. The biological admission of bone marrow concentrate (BMC) was repeated four times, and PRP treatments were administered eight times over a 24-month period. PRP intake in a torn medial collateral ligament because of an anatomical compartment leads to a proliferation phase of collagen production that results in the remodeling and restoration of strength and function. In a torn ACL, PRP is difficult to use because of anatomic locations without natural compartments, so clot formation never initiates and subsequent healing does not occur, so the repair procedures are ineffective. The intra-articular ACL does not heal because of a lack of wound site tissue bridging [16,17].

Murray et al. [51] proposed an alternative to ACL reconstruction: an operative method of ACL healing with a specific extracellular matrix scaffold that, in an intraoperative procedure, is placed in between ACL stumps to facilitate ligament healing. According to researchers, many patients are unable to return to preinjury levels of sports participation by 12 months post-ACL reconstruction. Further research and clinical outcomes proved that the presented method—bridge-enhanced anterior cruciate ligament repair—has noninferior patient-reported outcomes when compared with autograft anterior cruciate ligament reconstruction.

No operative procedure requirement and no graft intake lead to differences in clinical outcomes, including decreased pain levels and increased hamstring and quadriceps strength, which is crucial in the NSBT method [16,17,18].

The use of NSBT treatment for ACL injuries in my clinical practice significantly shortens patient recovery time compared to operative methods. In the future, NSBT treatment may significantly reduce the percentage of reconstructive procedures and may also reduce all the risk factors associated with hospitalization and operative methods.

This study has a limitation. Although the follow-up in the study group was 54.5 months, we did not compare the treatment group to a patient group that underwent ACL reconstructive surgery, but this is a reason to undertake another study. Another limitation is the lack of stratification based on sex and age. Nonetheless, the surgical treatment outcomes remain consistent across sexes and among patients aged 17 to 40 years.

## 5. Conclusions

This novel nonsurgical method was shown to be repeatable, objective, well-documented, standardized, and highly effective in the treatment of ACL tears, offering patients a rapid recovery. Importantly, to achieve good outcomes for NSBT application in patients with ACL tears, clinicians must strictly follow the guidelines for patient enrollment based on clinical evaluation, imaging studies, and nanosurgery procedures guided by ultrasonography. The findings of this study may represent a breakthrough in the novel treatment of patients with ACL tears.

## Figures and Tables

**Figure 2:**
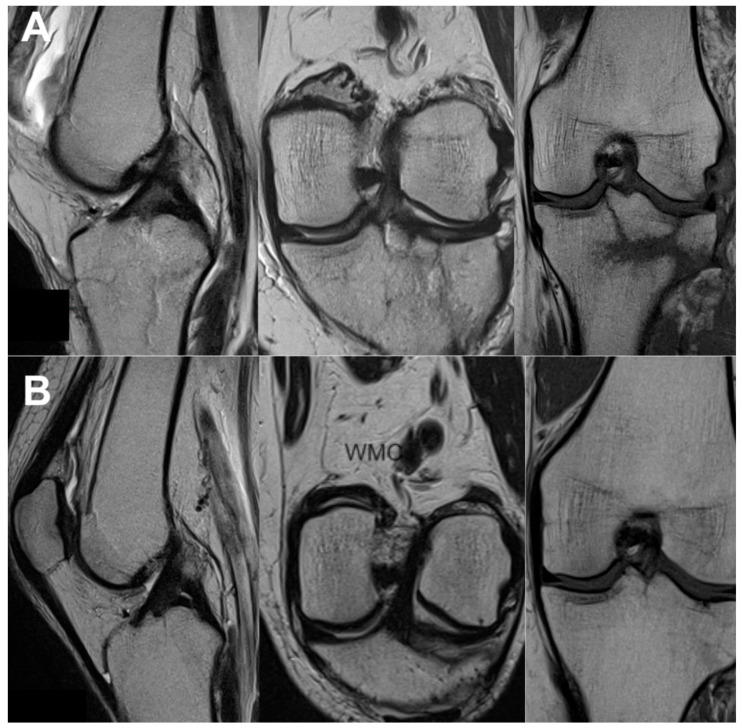
The same knee MRI scans of a patient with an anterior cruciate ligament tear and concomitant lateral tibia condyle fracture before treatment (**A**) and corresponding MRI scans of the same patient after nanosurgery treatment with an RP-hCM ACL injection (**B**). The time to return to regular daily activities with full weight-bearing on the injured limb without instability and pain was 9 weeks.

**Figure 3:**
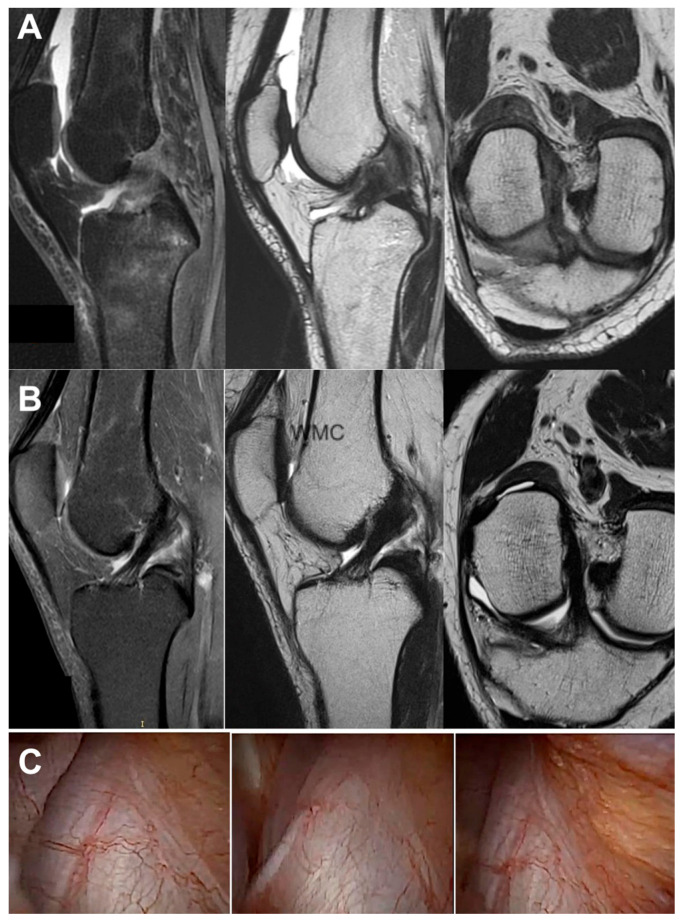
The same knee MRI scans of a patient with an anterior cruciate ligament tear before treatment (**A**) and corresponding MRI scans after nanosurgery treatment with an RP-hCM injection (**B**). A nanoscopic view of the normal, healed ACL (**C**).

**Figure 4:**
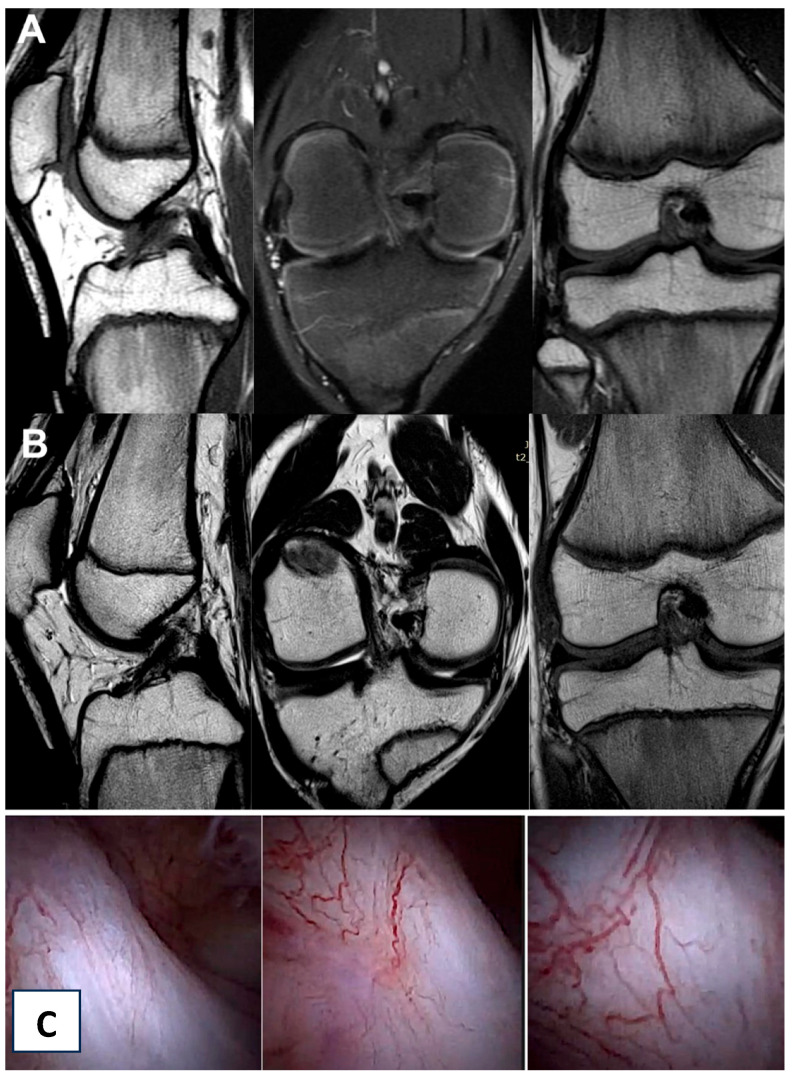
The same knee MRI scans of a patient with an anterior cruciate ligament tear before treatment (**A**) and corresponding MRI scans after nanosurgery treatment with RP-hCM injection (**B**). A nanoscopic view of the normal, healed ACL (**C**).

**Figure 5:**
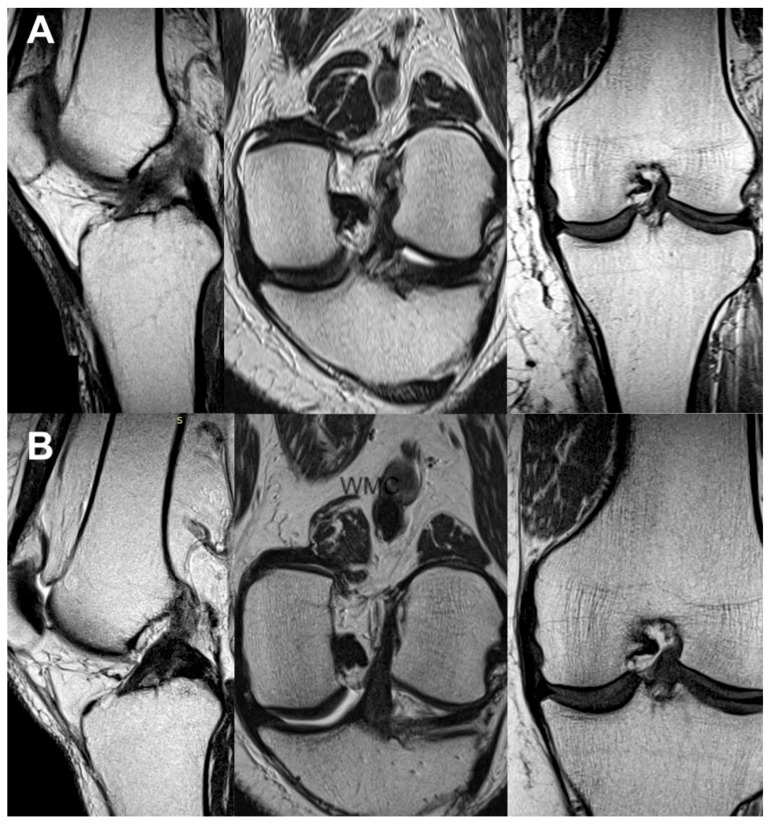
The same knee MRI scans of a patient with an anterior cruciate ligament tear before treatment (**A**) and corresponding MRI scans after nanosurgery treatment with an RP-hCM injection (**B**).

**Figure 6:**
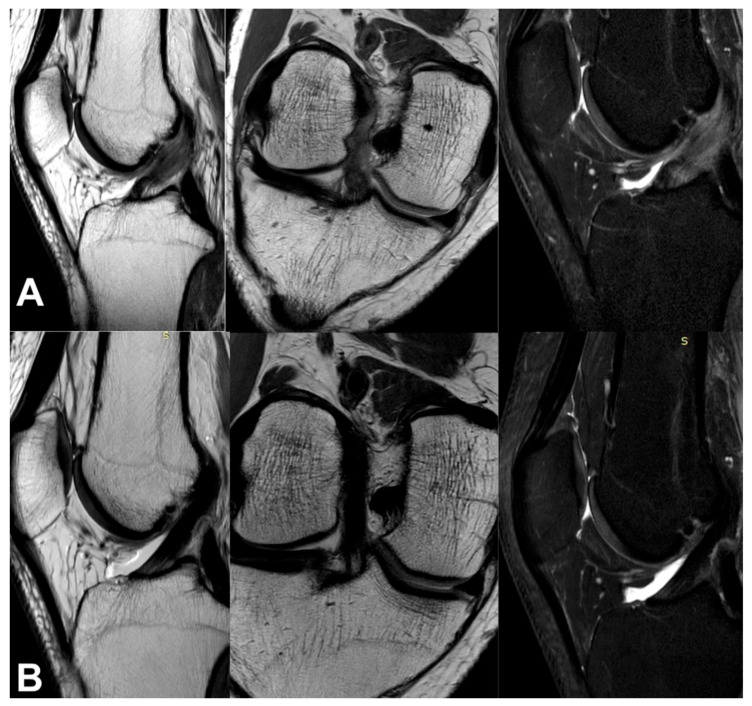
The same knee MRI scans of a patient with an anterior cruciate ligament tear before treatment (**A**) and corresponding MRI scans after nanosurgery treatment with an RP-hCM injection (**B**).

**Figure 7:**
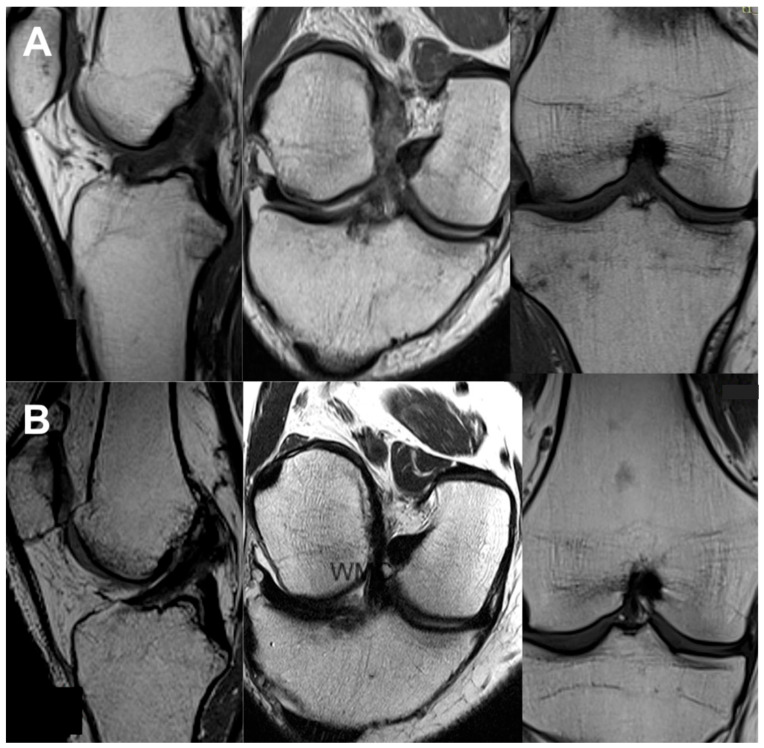
The same knee MRI scans of a patient with an anterior cruciate ligament tear before treatment (**A**) and corresponding MRI scans after nanosurgery treatment with an RP-hCM injection (**B**).

**Figure 8:**
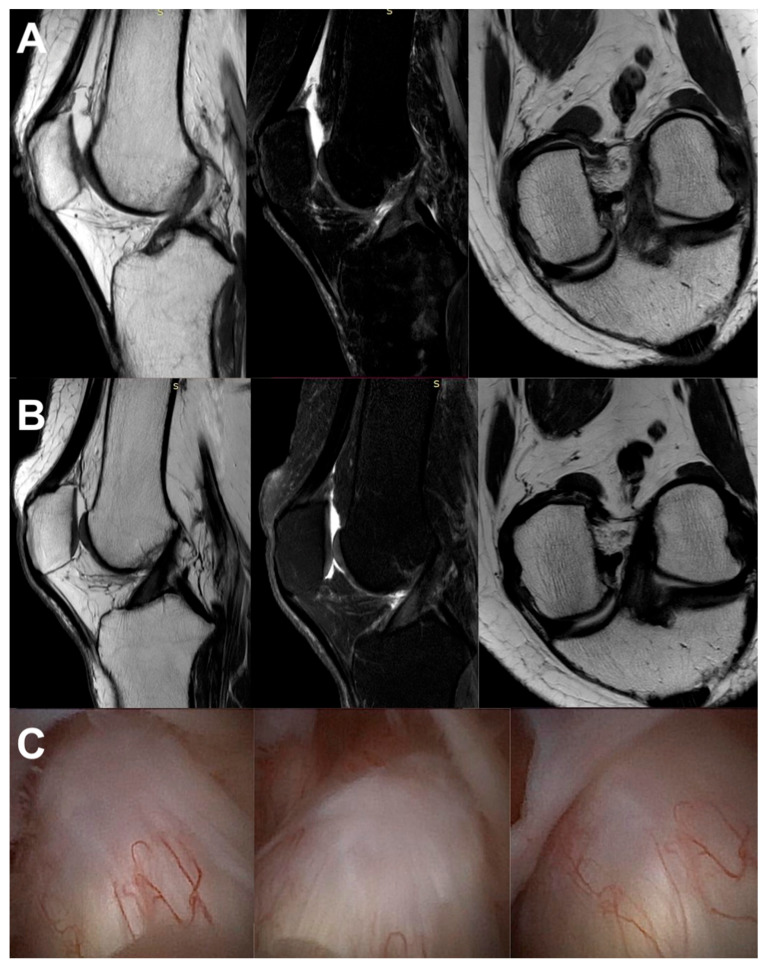
The same knee MRI scans of a patient with an anterior cruciate ligament tear before treatment (**A**) and corresponding MRI scans after nanosurgery treatment with an RP-hCM injection (**B**). A nanoscopic view of the normal, healed ACL (**C**).

**Figure 9:**
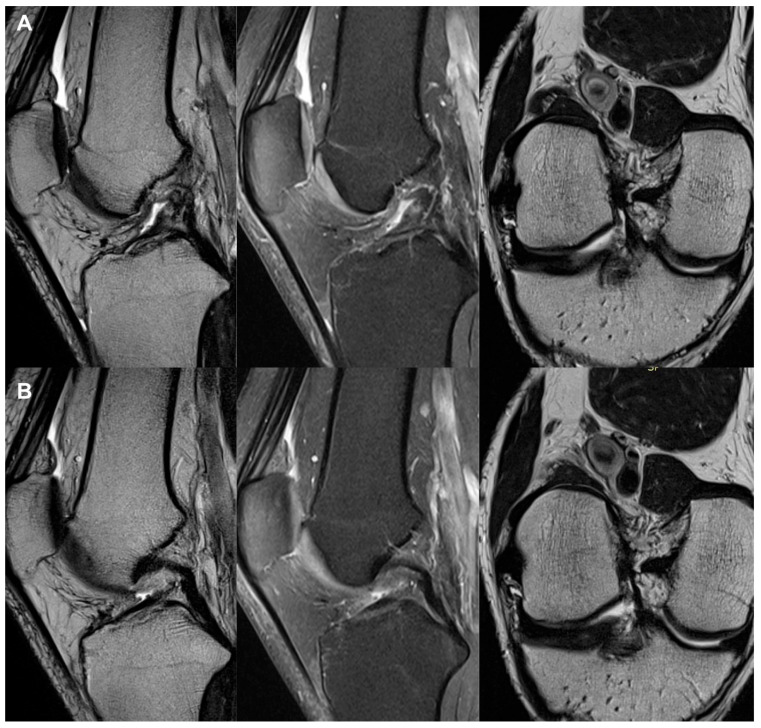
The same knee MRI scans of a patient from the control group with an anterior cruciate ligament tear before treatment (**A**) and corresponding MRI scans after treatment with PRP injection (**B**).

**Table 1:** Baseline characteristics of the study population.

Variable		Treatment Group (n = 30)	Control Group (n = 10)	*p*-Value
Age, years, median (IQR)		46.5 (15.0)	33.0 (12.0)	0.033
Sex, n (%)	Men	16 (53.3%)	9 (90%)	0.040
	Women	14 (46.7%)	1 (10%)	
BMI, mean (SD)		23.7 (2.3)	24.2 (1.1)	0.523
Injured knee, n (%)	Right	8 (26.7%)	4 (40%)	0.420
	Left	22 (73.3%)	6 (60%)	
Nanoscopy, n (%)	Yes	17 (56.7%)	1 (10%)	0.011
	No	13 (43.3%)	9 (90%)	

BMI—body mass index; IQR—interquartile range

**Table 2:** WOMAC, VAS, LKS, and physical examination scores at baseline and 12 weeks after treatment in the treatment and control groups.

Variable		Treatment Group (n = 30)	Control Group (n = 10)	*p*-Value
WOMAC, median (IQR)	At baseline	90.0 (8.0)	85.5 (10.0)	0.254
	After treatment	0 (0)	53.0 (10.0)	<0.001
	Change	−88.0 (66.0)	−30.0 (9.0)	<0.001
VAS, median (IQR)	At baseline	8.0 (1.0)	8.0 (0)	0.012
	After treatment	0 (0)	5.0 (1.0)	<0.001
	Change	−8.0 (7.0)	−3.0 (4.0)	<0.001
LKS, median (IQR)	At baseline	100 (0)	100 (2.0)	0.165
	After treatment	0 (0)	66.5 (15.0)	<0.001
	Change	−100 (69.0)	−33.0 (21.0)	<0.0001
Knee instability, median (IQR)	At baseline	8.0 (1.0)	8.0 (0)	0.044
	After treatment	0 (0)	6.5 (1.0)	<0.001
	Change	−8.0 (8.0)	−2 (2.0)	<0.001

LKS—Lysholm knee score; IQR—interquartile range, VAS—visual analogue scale.

## Data Availability

The datasets used and/or analyzed during the current study are available from the corresponding author upon reasonable request.
